# Anaphylaxis after Shrimp Intake in a European Pediatric Population: Role of Molecular Diagnostics and Implications for Novel Foods

**DOI:** 10.3390/children10101583

**Published:** 2023-09-22

**Authors:** Michele Miraglia del Giudice, Giulio Dinardo, Angela Klain, Elisabetta D’Addio, Chiara Lucia Bencivenga, Fabio Decimo, Cristiana Indolfi

**Affiliations:** Department of Woman, Child and of General and Specialized Surgery, University of Campania Luigi Vanvitelli, 80138 Naples, Italy; michele.miragliadelgiudice@unicampania.it (M.M.d.G.); angela.klain@studenti.unicampania.it (A.K.); elisabetta.daddio1@studenti.unicampania.it (E.D.); chiaralucia.bencivenga@studenti.unicampania.it (C.L.B.); fabio.decimo@unicampania.it (F.D.); cristiana.indolfi@unicampania.it (C.I.)

**Keywords:** food allergy, food hypersensitivity, shrimp, der p 10, tropomyosin, atopy, children, edible insect, insect flour

## Abstract

(1) Background: Tropomyosin is a major cause of shellfish allergy and anaphylaxis triggered by food. It acts as a pan-allergen, inducing cross-reactivity in insects, dust mites, crustaceans, and mollusks. Our study investigates anaphylaxis in children with asthma or atopic diseases after consuming tropomyosin-containing food. (2) Methods: We analyzed the molecular sensitization profiles of pediatric patients at the University of Campania ‘Luigi Vanvitelli’ from 2017 to 2021, with conditions such as allergic rhinitis, asthma, atopic dermatitis, urticaria, and food allergies. (3) Results: Out of a total of 253 patients aged 1 to 18 years (167 males, 86 females), 21 patients (8.3%) experienced anaphylaxis after shrimp ingestion. All 21 (100%) were sensitized to various tropomyosins: Pen m 1 (100%), Der p 10 (90.5%), Ani s 3 (81%), and Bla g 7 (76.2%). Clinical symptoms included allergic asthma (76.2%), atopic dermatitis (61.9%), urticaria (38.1%), and allergic rhinitis (38.1%). (4) Conclusions: Crustaceans and mollusks are major allergens in Italy and Europe, requiring mandatory declaration on food labels. Italian pediatric patients demonstrated significant anaphylaxis after consuming shrimp, often accompanied by multiple atopic disorders such as asthma, rhinitis, and atopic dermatitis. Considering the cross-reactivity of tropomyosin among various invertebrates and the emergence of ‘novel foods’ containing insect flours in Europe, there is ongoing debate about introducing precautionary labeling for these products.

## 1. Introduction

**Figure 1:**
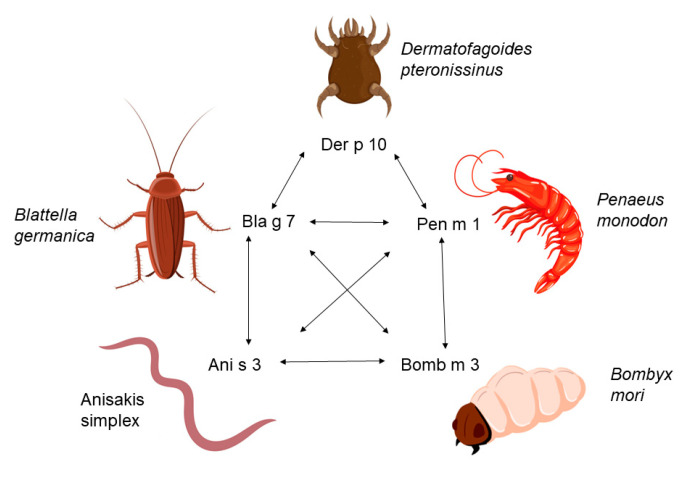
Cross-reactivity of tropomyosins among invertebrate species.

Shellfish is a common source of food-induced anaphylaxis and one of the most common lifelong allergies [1]. Up to 10% of the population suffers from shellfish allergy, particularly in Asia and the Pacific regions [2,3,4,5]. In the United States, an epidemiological study showed a frequency of shellfish allergy of about 2.2%, with a prevalence of 0.5% in children [6]. Wong et al. indicated that children’s self-reported rates of shellfish allergy are from 0.06 to 2% in occidental countries [7]. Airborne allergens are particularly abundant close to the cooking of shellfish by boiling, steaming, or frying. In 10% of cases, clinical reactions occur only at contact or through inhalation. It is notable that in many patients, clinical reactions after inhalation of shellfish were more severe than those induced by oral intake [8]. Workers in the seafood industry are more susceptible to shellfish allergy. It is estimated that 7% of workers with ingestion-related seafood allergy also experience asthma symptoms due to inhaling seafood [9]. IgE-mediated allergic reactions to shellfish can be immediate or delayed for up to 8 h after consumption, and they can affect one or more organs. The most frequently reported reactions are oral symptoms, including itching in the mouth, throat, and lips. Urticaria, periorbital angioedema, and skin redness are examples of cutaneous manifestations that follow oral food challenge-related reactions [10]. Although other allergens may play a significant part in allergenicity such as arginine kinase and myosin light chain [11], the major shellfish allergen is tropomyosin [12]. Tropomyosin belongs to a family of proteins associated with the thin filament in muscle and typically comprises a pair of alpha-helical tropomyosin molecules, which intertwine with each other to form a coiled-coil dimer structure. Together with actin and myosin, tropomyosin plays a functional role in the contractile activities of muscular cells. One notable characteristic of tropomyosin is its high degree of amino acid sequence similarity within these molecules [13]. Tropomyosin is a pan-allergen which causes cross-reactivity between crustaceans, mites, insects, and nematodes [14]. Tropomyosin is the primary allergenic molecule for crustaceans and mollusks and has been recognized as a prominent allergen in various organisms. These include the house dust mite (Der p 10), crustaceans (Pen m 1), moths (Bomb m 3), and cockroaches (Bla g 7) [15]. Additionally, tropomyosin is found in the herring worm *Anisakis simplex* (Ani s 3) and the common roundworm *Ascaris lumbricoides* (Asc l 3) (Figure 1) [16].

Tropomyosin can cause first sensitization through the gastrointestinal system (in this case, the allergen is typically associated with crustaceans or mollusks), or through the respiratory system (the allergen is typically associated with mites, Blattella, and insects). Initial sensitization in every case may lead to cross-reactivity to other related tropomyosins [13]. Tropomyosins share high structural homology and consequent IgE co-reactivity. Recently, it has been proven that tropomyosin T-cell cross-reactivity, unlike IgE cross-reactivity, is dependent on structural protein stability, such as the proteolytic digestion, generating different peptide collections [17]. In the 1980s, Hoffman identified the shrimp tropomyosin allergen (Pen a 1) [18]. Pen a 1 is a shrimp muscle protein tropomyosin of 36 kDa and is responsible for most of the allergenic activity of shrimp [13]. When compared to the skin prick test (SPT) for whole shrimp extract, the serum-specific IgE measurement for shellfish tropomyosin sensitization had a better sensitivity and specificity for predicting shellfish allergy [19,20]. Der p 10 is a 36 kDa shrimp muscle protein tropomyosin found in the house dust mite (HDM). Der p 10 shares high sequence homology with Pen a 1, with an amino acidic sequence similarity of 81% and four identical IgE binding epitopes [21,22]. Allergen Pen m 1 is the tropomyosin of the *Penaeus monodon*, also known as the Asian tiger shrimp, and it shares 99% identity with Pen a 1 [23]. Cockroach tropomyosins, Bla g 7 and Per a 7, also share structural homology with shrimp tropomyosin at 82%, while *Anisakis simplex* Ani s 3 and the terrestrial snail tropomyosin, *Helix aspersa* Hela a 1 have structural similarities of 74% and 61%, respectively [24]. *Anisakis simplex*, a common fish parasite, can be a hidden food allergen, inducing IgE-mediated reactions. The role of tropomyosin in the allergic cross-reactivity between this nematode and HDMs, cockroaches, shellfish, and edible insects has been confirmed [16,25,26]. In a study by Broeckman et al., we observed that shrimp-allergic patients with a food allergy to mealworms exhibited IgE reactivity by basophil activation test (BAT), ImmunoCAP Specific IgE test, and Western blotting to all insect extracts, and the primary IgE binding proteins were tropomyosin and/or arginine kinase [27]. Europe is known for its significant consumption of various seafoods, including crustaceans and mollusks. In fact, both crustaceans and mollusks, due to their high consumption in certain Europe regions and their potential to induce clinical reactions, are among the 14 major allergens that must, by law, be declared on food labels within the European Union [28,29]. However, it is important to acknowledge that the significance of these allergens can vary considerably based on the dietary habits and regional differences within European countries. As highlighted in recent research, the importance of specific allergens such as shrimp and fish in causing food allergies can vary geographically [30]. For instance, studies have shown that shrimp and fish were significant contributors to probable food allergies in regions such as Madrid (shrimp and fish), Athens (fish), and Reykjavik (shrimp and fish). However, this trend was not observed in other parts of Europe [31]. The EU Commission recently authorized the introduction of yellow mealworm flour (*Tenebrio molitor*) and cricket flour (*Acheta domesticus*) as “novel foods” into the European food market. Novel foods are defined as those that were not commonly consumed within the EU before May 1997 [32]. The recent introduction of these insect flours into the European food market raises particular concerns due to the allergic cross-reactivity between shrimp and edible insects such as mealworms, highlighting the need for potential precautionary measures to be adopted in food labeling Our study aims to evaluate the prevalence of sensitization to tropomyosin within a European pediatric population and the frequency of anaphylactic reactions after the consumption of crustaceans or mollusks in a pediatric population sensitized to tropomyosin. Anaphylaxis in children can manifest rapidly and severely, affecting the respiratory, cardiovascular, and cutaneous systems, demanding immediate medical attention [33]. This emphasizes the critical importance of understanding the prevalence of shellfish allergy, sensitization profiles, and potential cross-reactivity among different tropomyosins in pediatric populations. Furthermore, we explore the potential cross-reactivity among different tropomyosins and their association with allergic manifestations. The study of the prevalence of sensitization to tropomyosins within a European pediatric population could be crucial for a deeper understanding of their molecular sensitization profile. This knowledge can help in making informed choices and safeguarding consumers’ health.

## 2. Materials and Methods

### 2.1. Patients

Pediatric patients of both sexes ranging in age from one to eighteen years at the pediatric allergology clinic of the University of Campania ‘Luigi Vanvitelli’ over a period from 2017 to 2021 who had taken an ImmunoCAP ISAC test were selected. The patients under study were being followed for atopic disorders such as allergic asthma, atopic dermatitis, urticaria, allergic rhinitis, and food allergies. We excluded from the study patients with a history of severe underlying medical conditions, such as chronic systemic diseases or immunodeficiency. Additionally, patients who were currently undergoing treatment with biological drugs were also excluded from the study.

### 2.2. Study Design

Molecular sensitization profiles of the patients were retrospective analyzed using the microarray method (ImmunoCAP ISAC, ThermoFisher Scientific, Uppsala, Sweden). The ImmunoCAP ISAC method utilizes a solid-phase fluorescence immunoassay microarray system. It detects IgE antibodies directed against specific proteins immobilized on the ISAC surface. The test involves incubating each microarray with a serum sample to label specific IgE antibodies to the proteins of interest. Afterward, a human anti-IgE detection antibody conjugated with a fluorescent marker is applied. The fluorescence intensity of each microarray is quantified using a scanner with specific parameters. The results are expressed semi-quantitatively as ISUs (ImmunoCAP Standardized Units). Any results equal to or greater than 0.30 ISU-E are considered positive, following the manufacturer’s recommendations [34].

We analyzed sensitization to 4 different arthropoda and nematoda tropomyosins (Pen m 1 *Penaeus monodon*; Der p 10 *Dermatophagoides pteronyssinus*; Bla g 7 *Blattella germanica*; and Ani s 3 *Anisakis simplex*) included within the ImmunoCAP ISAC. Clinical data of patients such as the presence of asthma, atopic dermatitis, rhinitis, and urticaria and the history of anaphylaxis after food ingestion were evaluated. To diagnose food-induced anaphylaxis, we followed the guidelines provided by the European Academy of Allergy and Clinical Immunology (EAACI) for anaphylaxis diagnosis [33].

These data regarding molecular sensitization, clinical data, and laboratory data were compared to assess differences within the population. Our study was a retrospective study conducted in accordance with the Declaration of Helsinki and was approved by the Institutional Ethics Committee of University of Campania “Luigi Vanvitelli”.

### 2.3. Endpoint

The primary endpoint was to assess the prevalence of anaphylaxis after ingestion of shrimp or shellfish in an Italian pediatric population. The secondary endpoint was to evaluate the cross-reactivity of tropomyosins Der p 10, Pen m 1, Bla g 7, and Ani s 3 and to study the characteristics of patients with anaphylaxis after ingestion of shrimp or shellfish.

### 2.4. Statistical Analysis 

The patients’ characteristics were determined using descriptive statistics and are presented as a percentage. We compared the data obtained on the clinical and molecular sensitization profiles analyzed during the study using the chi-square test. Significance was set for *p*-values < 0.05. All the analyses were performed using Microsoft Excel for Microsoft 365, Microsoft Inc, Redmond, Washington, DC, USA and GraphPad Prism version 8.0.2 for Windows, GraphPad Software, San Diego, CA, USA.

## 3. Results

**Figure 2:**
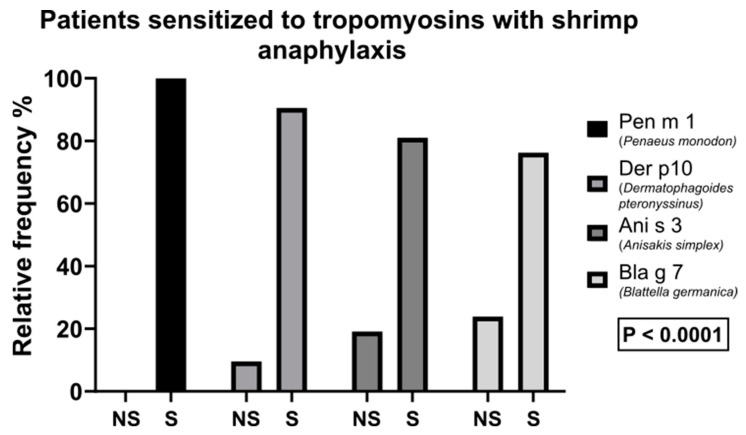
Patients sensitized to tropomyosins with shrimp anaphylaxis. NS: non-sensitized; S: sensitized.

A total of 253 patients were analyzed in the study, 167 males and 86 females, with a mean age of 150.42 months (min 53 months; max 213 months). A total of 21 (8.3%) patients had a clinical history of anaphylaxis following the ingestion of shrimp or shellfish. The mean age of patients with a clinical history of anaphylaxis after consuming shrimp or shellfish was 122.52 months (minimum 53 months; maximum 213 months). Acute episodes of anaphylaxis develop within minutes following the food ingestion. These episodes are characterized by involvement of the skin and mucosal tissues, including hives, itching, swelling of the lips and tongue, and respiratory compromise (dyspnea, wheezing, and bronchospasm). A total of 23 (9.1%) patients were sensitized to Der p 10; 31 (12.3%) patients were sensitized to Pen m 1; 19 (7.5%) patients were sensitized to Ani s 3; and 20 (7.9%) patients were sensitized to Bla g 7 (7.9%). (Table 1) The analysis of the data regarding the population with a clinical history of anaphylaxis after ingestion of shrimp or shellfish showed that all 21 (100%) patients were sensitized toward Pen m 1, 19 (90.5%) patients toward Der p 10, 17 (81%) patients toward Ani s 3, and 16 (76.2%) toward Bla g 7 (Figure 2).

**Figure 3:**
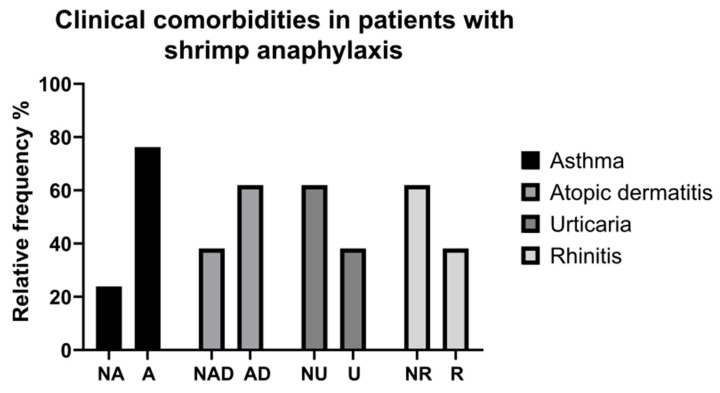
Clinical comorbidities in patients with shrimp anaphylaxis. NA: non-asthmatics; A: asthmatics; NR: non-rhinitic; R: rhinitic; NAD: non-dermatitic atopic; AD: atopic dermatitis; NU: non-horticarial; U: horticarial.

Moreover, of this group of patients, sixteen (76.2%) suffered from allergic asthma, thirteen (61.9%) from atopic dermatitis, eight (38.1%) from urticaria, and eight (38.1%) from allergic rhinitis (Figure 3). In our study, we performed a chi-square test to investigate the relationship between sensitization to tropomyosins and the occurrence of anaphylaxis subsequent to the consumption of crustaceans and mollusks. The results demonstrated a highly significant association between these variables, with a *p*-value of less than 0.0001, indicating strong statistical significance (Table 2). 

## 4. Discussion

Our study investigated the relationship between shellfish/shrimp allergy and tropomyosin sensitization in children based on the analysis of 253 patients (167 males and 86 females). It is important to note the distinction between sensitization and clinical allergy. While a proportion of patients exhibited sensitization to tropomyosins, not all of them experienced overt clinical allergic reactions upon exposure to shrimp or shellfish. Sensitization refers to the presence of specific IgE antibodies against an allergen, indicating an immune response to the substance. Clinical allergy, on the other hand, involves the manifestation of symptoms upon allergen exposure, which can range from mild to severe, such as anaphylaxis. In our study, we found that 8.3% of patients had a clinical history of anaphylaxis after consuming shrimp or shellfish. Among the studied tropomyosins, Der p 10, Pen m 1, Ani s 3, and Bla g 7, the sensitization rates were as follows: 9.1%, 12.3%, 7.5%, and 7.9%, respectively. Remarkably, all 21 patients with a history of anaphylaxis after ingesting shrimp or shellfish were sensitized to Pen m 1, indicating a strong association between this tropomyosin and severe allergic reactions. Additionally, 90.5% of these patients showed sensitization to Der p 10, 81% to Ani s 3, and 76.2% to Bla g 7. These findings highlight the potential cross-reactivity between various tropomyosins and suggest that Pen m 1 might play a crucial role in triggering severe allergic responses in pediatric patients with shrimp/shellfish allergy. 

There are several studies in the literature that have evaluated different aspects of cross-reactivity between shrimp and other invertebrates [30]. The data from skin prick tests (SPTs) report an elevated rate of sensitization to HDMs in shrimp/shellfish-allergic patients [35]. In Chiang et al.’s study, 72% of shellfish-sensitized Asian individuals had a SPT positivity to HDMs [36]. In an Ontario population, 90.5% of 95 shrimp-allergic patients had a positive SPT to HDMs [37]. In addition, shrimp IgEs of the shrimp-sensitized patients from rural areas were associated with sensitization to either dust mites or cockroaches (*p* < 0.001), revealing that for certain rural children with shrimp sensitivity, cockroaches may be the primary sensitizer allergen. Additionally, cockroach allergy may impact the clinical importance of shrimp allergy in rural regions [38]. Thus, it appears that exposure to mites or cockroaches in the environment and the resulting sensitization to these arthropods may drive shellfish sensitization [39,40,41]. Indirect evidence from the effects of HDM immunotherapy on shellfish allergy as well as significant correlations between shellfish and HDM sensitization support this idea. IgE and immunoblot inhibition studies highlight a strong correlation between IgE to shrimp and HDM tropomyosins, with an almost complete inhibition of shrimp extract by mites, suggesting that HDMs are the primary sensitizers [39,42]. HDM immunotherapy has been documented to induce both shrimp allergy in non-allergic patients and shrimp tolerance in shrimp-allergic patients [43,44]. Furthermore, our study demonstrated a high prevalence of atopic symptoms, such as allergic asthma, atopic dermatitis, urticaria, and allergic rhinitis, in pediatric patients sensitized against tropomyosin. Specifically, 76.2% of shellfish/shrimp-allergic children had asthma, 61.9% had atopic dermatitis, 38.1% experienced urticaria, and 38.1% suffered from allergic rhinitis. The symptoms of shellfish/shrimp allergy have so far mostly been described in relation to fishermen or other workers in contact with shellfish. Increased levels of seafood production and processing lead to more frequent reporting of occupational health problems such as rhinitis, urticaria, asthma, and other allergic reactions [45]. Between 2% and 36% of shellfish processors are estimated to suffer from occupational asthma, whereas the rate of occupational dermatitis is 3–11% [46]. In general, food allergy is a recognized risk factor for developing asthma, with an odds ratio of 2.16 [47]. Roberts et al. reported that children with food allergies, in particular, egg or milk in the first years of life, were around six times more likely to develop severe asthma later in life than children who did not have food allergies [48]. This evidence is in line with the concept of atopic march [49,50]. Wang et al. demonstrated that children with shellfish allergy are more likely to be black or Hispanic/Latino and more likely to suffer from asthma and allergic rhinitis as well as a family history of asthma, environmental allergies, or other food allergies (*p* < 0.001) compared to the general American pediatric population [51]. The flipside of the same coin is that shellfish allergy and asthma co-existence is associated with an increased risk of anaphylaxis [7].

Interestingly, no other European research focusing on a pediatric population with shrimp/shellfish anaphylaxis was found in the literature, which emphasizes the importance of sensitization to Der p 10 and other tropomyosins through Component-Resolved Diagnosis (CRD). Additionally, our findings align with other studies that have highlighted the link between mites or cockroaches and other invertebrates in subsequent shellfish sensitization. This suggests that cross-reactivity with HDMs and other arthropods may drive shellfish sensitization, possibly explaining the higher rates of sensitization to HDMs observed in shrimp/shellfish-allergic patients.

Our study has focused on evaluating sensitization to tropomyosin from various invertebrate species, as tropomyosin remains the primary target for immunodiagnostic and therapeutic advancements in shellfish/shrimp allergy. Clinically, avoiding foods containing shrimp can help patients with shrimp allergy avoid further sensitization and IgE-mediated reactions, but it is unknown whether environmental exposure to HDMs or insect-derived tropomyosin or the fish parasitic nematode Anisakis simplex tropomyosin could result in further priming and the re-stimulation of a shrimp-specific allergic response [17]. The role of Component-Resolved Diagnosis (CRD) in individuals with tropomyosin sensitization could be crucial for making informed dietary choices and constitutes an important field of research. The molecular cross-reactivity of allergic patients is based on the IgE identification of identical and/or comparable homologous peptides to the allergen epitopes [52]. A recent Australian study found that comparing specific epitopes to the entire protein sequence of tropomyosin is more useful for predicting allergic reactions to ingested crustaceans in patients allergic to cockroaches and HDMs as well as for diagnosing cross-reactivity between crustacean and mollusk species in shellfish-allergic patients [53]. According to latest EAACI molecular allergology user guide, due to the cross-reactivity of tropomyosin and arginine kinases, there is a high probability that individuals allergic to shrimp will also exhibit reactions to different types of edible insects [16]. The current knowledge regarding the allergenic properties of edible insects remains quite restricted. Broekman et al. observed that out of 15 shrimp-allergic patients, 14 were sensitized to mealworm tropomyosin or arginine kinase and demonstrated through a double-blind, placebo-controlled food challenge that 13 out of 15 shrimp-allergic patients reacted to a meal containing mealworm. The research also revealed that all 15 patients showed sensitization to mealworm extract, as determined by basophil activation test (BAT), ImmunoCAP Specific IgE test, and Western blotting. Among them, ten mealworm-allergic patients had IgE antibodies against tropomyosin, while three had IgE against arginine kinase. Furthermore, the patients showed specific IgE against the house cricket, giant mealworm, lesser mealworm, African grasshopper, large wax moth, and black soldier fly, and this specific IgE was capable of activating basophils. Both tropomyosin and arginine kinase were identified as the main allergens responsible for cross-reactivity between shrimp and insects [27,54]. These insects have the potential to trigger cross-reactivity with commonly consumed foods such as crustaceans, as well as with widely encountered invertebrate inhalant allergens such as house dust mites (HDMs). Given the phylogenetic relationship between insects and other arthropods, there is a legitimate concern in allergology that individuals with a shrimp allergy may experience allergic reactions upon consuming edible insects. In Western countries, it is not common to consume insect-based food. However, the recent introduction of insect-based flours in Europe raises the question of whether the proteins they contain can elicit allergic reactions. The impact of postharvest processing, heat processing, and the pasteurization process on the allergenic potency of edible insect proteins is still under investigation. It has been observed that enzymatic hydrolysis or heat treatment can reduce allergenicity in individuals allergic to crustaceans and house dust mites when consuming migratory locusts. Conversely, in another study, the heat treatment of fresh mealworms did not lead to a reduction in allergenicity among a group of 15 shrimp-allergic patients [55]. Unfortunately, knowledge about the impact of processing methods on insect allergenicity is still limited, and data available in the literature often show conflicting results. We hope that rigorous studies on this issue will be conducted in the future [5,16,56]. 

Tropomyosin remains a focal point for immunodiagnostic and therapeutic strategies in shellfish/shrimp allergy management. Clinically, the avoidance of foods containing shrimp can help prevent further sensitization and IgE-mediated reactions in patients with shrimp allergy [30]. However, given the rising popularity of insect-flour-based novel foods, additional research is needed to assess their potential impact on allergic responses, especially in individuals with a pre-existing shrimp allergy. 

Our study aims to provide further insights into the complex relationship between shellfish allergy, tropomyosin sensitization, and the potential risks associated with the consumption of novel foods containing insect flours in the European market. The prevalence of shellfish allergy is a significant concern, particularly in Asian and Pacific regions where up to 10% of the population suffers from this lifelong allergy. In contrast, the United States reports a lower frequency of shellfish allergy, around 2.2%, with even lower prevalence rates in children. Such variations in shellfish allergy prevalence emphasize the importance of understanding regional differences in dietary habits and allergenicity. One of the key findings of our study is the rate of sensitization to tropomyosin among the pediatric population in Italy. Tropomyosin, a major shellfish allergen, exhibits structural homology across various invertebrate species, leading to cross-reactivity. In our study, we observed that individuals sensitized to tropomyosin were at a significantly increased risk of experiencing anaphylactic reactions after ingesting crustaceans or mollusks. This highlights the need for precise diagnostic tools to identify tropomyosin sensitization and educate individuals at risk. Moreover, our study sheds light on the potential cross-reactivity between tropomyosins from different invertebrates, including crustaceans, mites, insects, and nematodes. For example, individuals with shrimp allergy may also react to mealworms due to shared allergenic proteins such as tropomyosin and arginine kinase. As “novel foods” containing insect flours, such as yellow mealworm and cricket flour, gain popularity in the European market, it becomes essential to investigate their allergenicity and potential impact on individuals with pre-existing shrimp allergies. Clear food labeling and informed choices are crucial to prevent accidental consumption and allergic reactions.

Limitations of our study include the lack of prospective data on the development of asthma and/or rhinitis in patients with shellfish/shrimp allergy sensitized to HDMs and/or other anthropoids. Therefore, longitudinal studies are warranted to explore these associations more comprehensively. Overall, our findings highlight the importance of further research in the field of tropomyosin sensitization and its implications for shellfish and insect allergies. 

Prospective studies are warranted to confirm and expand upon these results, as well as to investigate the underlying mechanisms of cross-reactivity between tropomyosins from different invertebrates. Furthermore, as “novel foods” containing insect flours are introduced into the European market, it is essential to conduct research to assess their allergenicity and potential impact on individuals with pre-existing shrimp allergies. Addressing these research gaps will enhance our understanding of allergenic cross-reactivity, assist in the development of precise diagnostic tools, and facilitate the implementation of effective precautionary measures to safeguard the health and well-being of individuals with allergies.

## 5. Conclusions

Currently, gaps exist in our understanding of the natural history of shellfish allergy in European children, as well as the roles of HDMs and/or other anthropoids in the development of primary allergen sensitization. In Italy and the rest of Europe, shrimp and shellfish are part of the category of foods that must be mandatorily highlighted within food labeling. Our study allowed us to observe that sensitization to tropomyosin is not uncommon in the Italian pediatric population. The presence of sensitization to tropomyosin correlates with an increased risk of anaphylactic reactions after the ingestion of shrimp or shellfish, with reactions also described after the inhalation of cooking vapors. In addition, this sensitization would also appear to correlate with increased atopic manifestations such as asthma and atopic dermatitis. The precise diagnosis of shellfish allergy poses a challenge due to the diversity of consumed species and the immunological cross-reactivity to similar invertebrate pan-allergens. Several studies have demonstrated that due to the cross-reactive nature of tropomyosin, shrimp allergy can also be indicative of allergies to insects such as mealworms. CRD can help us to have a better estimate of sensitized people and offer these people important information in case of clinical reactions after the intake of these foods. Furthermore, due to the cross-reactivity of tropomyosin among different invertebrates, there is particular concern regarding the presence of “novel foods” such as insect flours in the European market. In the absence of clearly marked precautionary food labelling, consumers may be unable to make safe and informed food choices. Accidental consumption of these types of food could potentially lead to significant allergic reactions. Moreover, familiarity with these allergens would confer significant benefits in conducting longitudinal studies aimed at assessing their potential cross-reactivity with other related food and inhalant allergens. By analyzing the IgE antibody recognition patterns across different groups, undertaking a follow-up investigation on the same or similar cohorts would contribute to the evaluation and validation of these shrimp allergens’ capacity to serve as early indicators of shrimp allergy.

## Figures and Tables

**Table 1:** Sensitization toward tropomyosins and patients with anaphylaxis from shrimp or shellfish in general population.

	Anaphylaxis	Pen m 1	Der p 10	Anis s 3	Bla g 7
Yes	21 (8.3%)	31 (12.3%)	23 (9.1%)	19 (7.5%)	20 (7.9%)
No	232 (91.7%)	222 (87.7%)	230 (90.6%)	234 (92.5%)	233 (92.1%)

**Table 2:** Sensitization toward tropomyosins in patients with and without anaphylaxis from shrimp or shellfish.

	Anaphylaxis: Yes	Anaphylaxis: No	*p*
Pen m 1 Median IgE (ISU-E)	21/21 7.3	4/232 0	<0.0001
Der p 10 Median IgE (ISU-E)	19/21 4.7	4/232 0	<0.0001
Bla g 7 Median IgE (ISU-E)	16/21 2.3	4/232 0	<0.0001
Ani s 3 Median IgE (ISU-E)	17/21 3.1	2/232 0	<0.0001

## Data Availability

The datasets used and/or analyzed during the current study are available from the corresponding author on reasonable request.
